# Glucose Starvation-Caused Oxidative Stress Induces Inflammation and Autophagy in Human Gingival Fibroblasts

**DOI:** 10.3390/antiox11101907

**Published:** 2022-09-26

**Authors:** Runbo Li, Hirohito Kato, Yoichiro Taguchi, Xin Deng, Emika Minagawa, Takaya Nakata, Makoto Umeda

**Affiliations:** Department of Periodontology, Osaka Dental University, Hirakata, Osaka 573-1121, Japan

**Keywords:** glucose, gingival fibroblasts, ROS, inflammation, autophagy

## Abstract

Gingival tissue experiences an environment of nutrient shortage, such as low glucose conditions, after periodontal surgery. Our previous studies found that this low glucose condition inhibits normal gingival cell functions. However, the mechanism by which this glucose-deficient environment causes cellular damage to human gingival fibroblasts (HGnFs) remains unclear. This study aimed to investigate the biological effects of ROS induction on HGnFs under low glucose conditions. ROS levels and cellular anti-ROS ability of HGnFs under different glucose concentrations were evaluated by measuring ROS formation and the expression of superoxide dismutase and heme oxygenase 1. Changes in cellular viability were investigated using 5-bromo-2′-deoxyuridine assay and cell survival detection, and the cellular damage was evaluated by the expression of inflammatory cytokines and changes in the expression of autophagy-related protein. ROS formation was then blocked using N-acetyl-L-cysteine (NAC), and the effects of ROS on HGnFs under low glucose conditions were investigated. Low glucose conditions induced ROS accumulation, reduced cellular activity, and induced inflammation and autophagy. After NAC application, the anti-ROS capacity increased, cellular activity improved, and inflammation and autophagy were controlled. This can be effectively controlled by the application of antioxidants such as NAC.

## 1. Introduction

Glucose is central to energy utilization [1]. Proteins, carbohydrates, and lipids are eventually catabolized into glucose, which then serves as a universal fuel for almost all major cellular metabolisms [2]. However, an excessive glucose condition can have toxic effects on the structure and function of cells [3]. Many studies have provided valuable information about the high glucose metabolism of cells leading to impaired cellular functions, including glucose autoxidation [4], activation of protein kinase C [5], formation and glycation of methylglyoxal, and oxidative phosphorylation [6]. Not only can glucose lead to toxicity, but excess glucose metabolites may also lead to cellular damage [7]. Common to all these pathways is the formation of reactive oxygen species (ROS), which can lead to impaired normal cell function, as well as increased cell death. Studies have shown that ROS directly or indirectly activates many signaling pathways in the cell, such as Akt and ERK, and this activation mechanism is one of the possible factors that induce cellular damage, such as inflammation [8].

Similar to all other mammalian cells, human gingival fibroblasts (HGnFs) utilize glycolytic pathways to maintain cellular function. However, under some conditions, if the current vessel system is damaged, cells experience a stressed environment characterized by low glucose concentrations. For example, following trauma or periodontal surgery, the blood supply to periodontal tissue is significantly lower than that to healthy tissue because of damaged blood vessels. We have found in our previous studies that low glucose conditions can inhibit normal gingival cell function, such as proliferation, migration, and collagen synthesis [9].

The Akt and ERK pathways reportedly collaborate to maintain cellular function [10]. These two signaling pathways are cross-linked at many sites, and either pathway can be signaled to enhance or inhibit the signaling of the other. For example, the ERK signaling pathway can be activated by phosphorylation, releasing growth factors that can feedback into the cell to activate the Akt signaling pathway. Both pathways can be activated by ROS and perform many physiological functions [8], such as immunological response and autophagy. Autophagy is a physiological process by which cells ensure their viability by securing a source of nutrients through self-digestion in the event of starvation [11]. Autophagy also induced by bacterial infection and inflammation can be detected in the gingival tissue [12,13]. Kim et al. have reported that autophagy could upregulate inflammatory cytokines in gingival tissue of patients with periodontitis, and lipopolysaccharide (LPS) could induce autophagy in gingival tissue [14]. Furthermore, autophagy is closely associated with inflammation [15]. Autophagy can influence the secretion of inflammatory cytokines and can be influenced by inflammatory cytokines. However, to the best of our knowledge, the relationship between ROS, inflammation, and autophagy in gingival tissues has not yet been investigated.

This study aimed to investigate the mechanisms of cellular damage in glucose starvation conditions and the relationship between ROS and the process of autophagy development. N-acetyl-L-cysteine (NAC) administration inhibited ROS formation under low glucose conditions, and inflammation and autophagy under such conditions were investigated. We hope that this study will lead to a better understanding of the cellular damage mechanism under low glucose conditions in wounded tissue.

## 2. Materials and Methods

### 2.1. Reagents and Antibodies

All chemical and biochemical reagents were purchased from the following sources: DMEM from Nacalai Tesque (Kyoto, Japan), FBS from Thermo Fisher Scientific (Rockford, IL, USA), and NAC (A7250) from Sigma-Aldrich (Burlington, MA, USA). The total ROS detection kit was obtained from Dojindo (Kumamoto, Japan). Antibodies against ERK, p-ERK, Akt, p-Akt, LC3B, p62, and β-actin were obtained from Cell Signaling Technology (Beverly, MA, USA). Antibodies against LC3 and p62 were obtained from MBL (Nagoya, Japan).

### 2.2. Cell Culture

HGnFs were obtained from ScienCell Research Laboratories (San Diego, CA, USA) and cultured in Dulbecco’s modified Eagle’s medium (DMEM) supplemented with 10% fetal bovine serum (FBS). The culture medium was changed every 3 days, and the cells were harvested and subcultured on reaching sub-confluence. The cells were used between passage numbers 3 and 6 for each experiment. HGnFs were incubated in DMEM with four glucose concentrations: 100 (normal control), 50, 25, and 0 mg/dL, and these were formed by mixing two kinds of DMEM with different glucose concentrations (100 and 0 mg/dL). The glucose concentrations of 100, 50, 25, and 0 mg/dL were equivalent to blood glucose levels of 5.5, 2.75, 1.375, and 0 mM, respectively, according to a previous study. Antioxidant treatment was performed by adding different concentrations (1, 5 and 10 mM) of N-acetyl-L-cysteine (NAC) at once to the 0 mg/dL group.

### 2.3. Detection of Cell Proliferation

Cell proliferation rates were determined by the uptake of 5-bromo-2′-deoxyuridine (BrdU, Nacalai Tesque) into DNA. The HGnFs were incubated in 100 mg/dL glucose until 80% confluence and were cultured under each glucose concentration for 24 h.

Next, a medium containing 10 μM BrdU was added to each well and incubated on plates for 2 h. Then, the cells were fixed and permeabilized. DNA was hydrolyzed with 2 M HCL. After nuclear staining with DAPI (Dojindo, Kumamoto, Japan), cells were observed under a Keyence BZ-II all-in-one fluorescence microscope (Keyence, Osaka, Japan). The proportion of BrdU-positive cells was determined by counting the 10 randomly captured BrdU-positive nuclei per well and the total nuclei.

### 2.4. Cell Survival Assay

Cell survival was measured using an MTT assay. The HGnFs were incubated until confluence, and the medium was changed to a medium containing different glucose concentrations (100, 50, 25, and 0 mg/dL). The data of viable cells at each time point were collected by measuring the amount of formazan using Cell Count Reagent SF (Nacalai Tesque, Japan). The data were analyzed using SoftMax^®^ Pro Microplate Data Acquisition and Analysis software (Version 7.0, Molecular Devices, Sunnyvale, CA, USA).

### 2.5. Immunofluorescence Staining

The HGnFs were seeded in 24-well microplates at 1 × 10^3^ cells/mL in a 1 mL culture medium and allowed to adhere for 24 h. The medium was replaced with a medium containing different glucose concentrations (100, 50, 25, and 0 mg/dL). Following 24, 72, or 120 h of incubation, the cells were fixed, permeabilized, and blocked, and then were incubated overnight at 4 °C with 250-times diluted, primary mouse, primary antibodies against LC3B and p62.

Fluorescent immunostaining was performed by applying Alexa Fluor 488^®^ (Thermo Fisher Scientific, Waltham, MA, USA) as the secondary antibody, and the nuclei were stained with DAPI (Dojindo). After staining, images were obtained using a BZ-II all-in-one fluorescence microscope and analyzed using ImageJ Ver. 1.53e (Wayne Rasband and contributors, National Institutes of Health, Bethesda, MD, USA, http://rsb.info.nih.gov/ij accessed on 10 December 2020.

### 2.6. ROS Detection and Staining

ROS levels were measured using a Total ROS Detection Kit (Dojindo), following the manufacturer’s instructions. The HGnFs were cultured to adhere for 24 h, and the medium was replaced with a medium containing different glucose concentrations (100, 50, 25, and 0 mg/dL). Then, the cells were incubated for 24, 72, or 120 h. The stained cells were observed and photographed using fluorescence microscope (Keyence). Cellular fluorescence was measured and analyzed using SoftMax^®^ Pro Microplate Data Acquisition and Analysis software.

### 2.7. Real-Time Polymerize Chain Reaction

The HGnFs were seeded and incubated for 24 h, and the medium was then replaced with a medium containing different glucose concentrations (100, 50, 25, and 0 mg/dL) and incubated for 24, 72, and 120 h.

The total RNA was isolated using the RNeasy Mini Kit (Qiagen, Venlo, The Netherlands) and reverse-transcribed to cDNA using a PrimeScript Reagent Kit (Takara Bio, Shiga, Japan). The gene expression of IL-6, IL-8, and IL-1β (Taqman gene expression assay) was quantified using a Step One Plus Real-Time PCR System (Applied Biosystems, Thermo Fisher Scientific, Waltham, MA, USA).

### 2.8. Human HO-1 and SOD ELISA

The HGnFs were seeded and allowed to adhere for 24 h. Then, the cells were cultured under each glucose concentration (100, 50, 25, and 0 mg/dL) for 24, 72, and 120 h. HO-1 and SOD secretion levels in culture supernatant were measured by using the Human Heme Oxygenase 1 ELISA Kit (ab207621, Abcam, Cambridge, UK) and the Human Superoxide Dismutase 1 ELISA Kit (ab119520, Abcam).

### 2.9. Western Blot Analysis

The HGnFs were treated with different glucose concentrations (100, 50, 25, and 0 mg/dL) for 5, 8, 24, 72, and 120 h and then harvested. The total protein was extracted using RIPA buffer (Thermo Fisher Scientific), supplemented with a protease inhibitor cocktail (Thermo Fisher Scientific) and a phosphatase inhibitor cocktail (Nacalai Tesque). The total protein concentration was determined using a BCA Protein Assay kit (Thermo Fisher Scientific, Waltham, MA, USA).

The protein samples were separated, transferred, and blocked. Then, the membranes (Bio-Rad, Hercules, CA, USA) were incubated with primary antibodies against ERK, p-ERK, Akt, p-Akt, β-actin, LC3B, and p62 (Cell Signaling Technology). Next, the membranes were washed and incubated with secondary antibodies (Cell Signaling Technology). Immunoreactive bands were visualized using a chemiluminescence kit (Nacalai Tesque). The signals and Western blotting data were analyzed using the ChemiDoc MP System (Bio-Rad). Statistical analyses were performed using ImageJ Ver. 1.53e (Bethesda, MD, USA).

### 2.10. Statistical Analysis

Data were presented as the mean ± standard deviation (SD). Parametric data were analyzed using one-way analysis of variance with Tukey’s test by IBM SPSS Statistics Ver. 17 (IBM, Chicago, IL, USA).

## 3. Results

### 3.1. Low Glucose Conditions Increase ROS Levels

**Figure 1:**
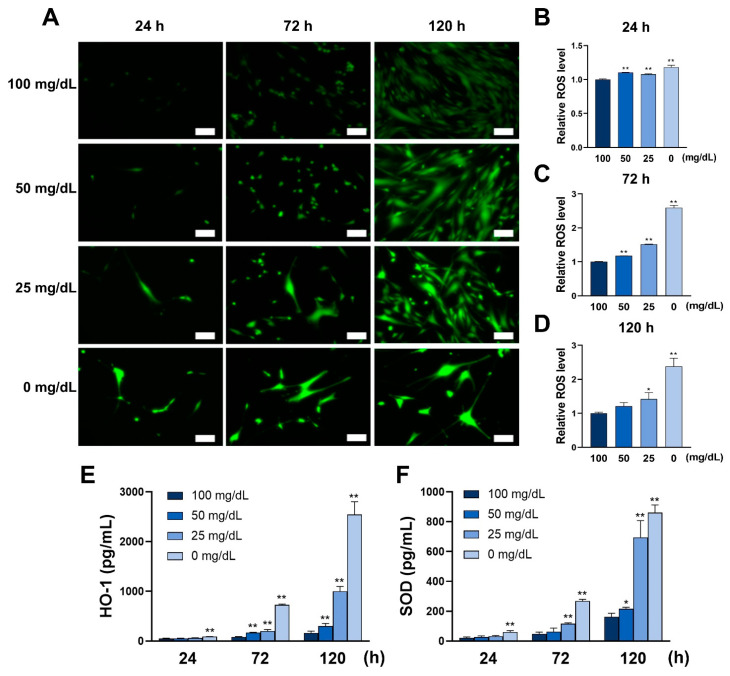
Low glucose conditions induce ROS by impairing the anti-ROS capacity of human gingival fibroblasts (HGnFs). (**A**) After 24, 72, and 120 h of incubation, the HGnFs were stained for total ROS evaluation using a ROS detection kit (Nacalai) and photographed under a fluorescent microscope. (**B**–**D**) The levels of total ROS at 24, 72, and 120 h were measured using a fluorescent microplate reader, and the results were modified relative to 100 mg/dL (control group). (**E**–**F**) The expression of SOD and HO-1 was determined using ELISA in supernatants of stimulated HGnFs incubated for 24, 72, and 120 h. (Scale bars: 100 μm; a significant increase compared with the control was described as * *p* < 0.05, ** *p* < 0.01).

The effects of four different glucose concentrations on ROS were measured after culturing for 24, 72, and 120 h. As shown in Figure 1, ROS levels increased significantly in all stimulation groups (50, 25, and 0 mg/dL), peaking at 120 h. The anti-ROS ability was measured by the secretion of HO-1 and SOD. The anti-ROS ability increased proportionately with the glucose concentration.

### 3.2. Low Glucose Conditions Impair Cellular Viability and Promote the Expression of Inflammatory Cytokines

Cellular viability was assessed by cell proliferation and survival. Cell proliferation and survival were reduced proportionately with different glucose concentrations after 24, 72, and 120 h of incubation.

**Figure 2:**
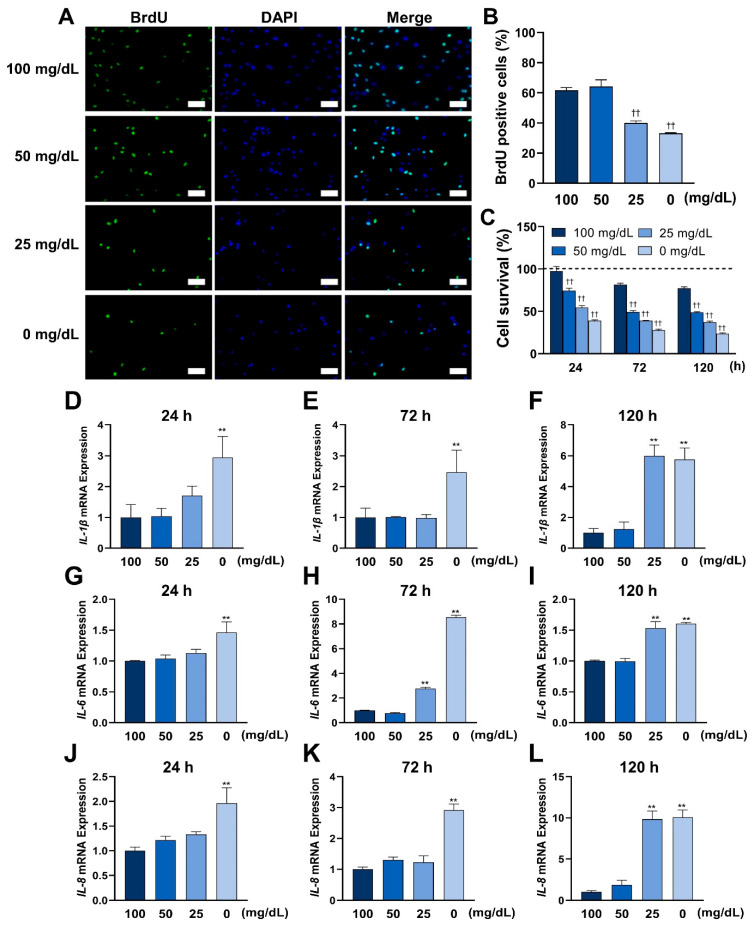
Low glucose conditions affect the cellular viability and promote the expression of inflammatory cytokines in HGnFs. (**A**,**B**) Representative immunofluorescence images of BrdU were incorporated into DNA, and the nuclei were stained by DAPI. The quantitative analysis of BrdU-positive cells was presented as mean ± SD of three independent experiments. (**C**) Cell survival was measured using an MTT assay. The average of the absorbance of each group at 0 h was used as 100% to calculate the relative results. (**D**–**L**) The mRNA expression of inflammatory cytokines, such as IL-1β, IL-6, and IL-8, was determined using real-time PCR in stimulated HGnFs incubated for 72 h. (Scale bars: 100 μm; a significant increase compared with the control was described as ** *p* < 0.01; a significant decrease compared with the control was described as †† *p* < 0.01.).

The effects of low glucose concentrations on the genes of inflammatory cytokines in HGnFs were analyzed using quantitative real-time PCR. As shown in Figure 2, the gene expression of IL-1β, IL-6, and IL-8 was significantly increased in the glucose 0 mg/dL group after 24 h of incubation, IL-6 expression increased in the glucose concentration 25 and 0 mg/dL groups after 72 h of incubation, IL-1β and IL-8 gene expressions increased in the glucose concentration 0 mg/dL group, and IL-1β, IL-6 and IL-8 gene expressions increased proportionally in the glucose concentration 25 and 0 mg/dL groups after 120 h of incubation.

### 3.3. Low Glucose Conditions Induce Autophagy

**Figure 3:**
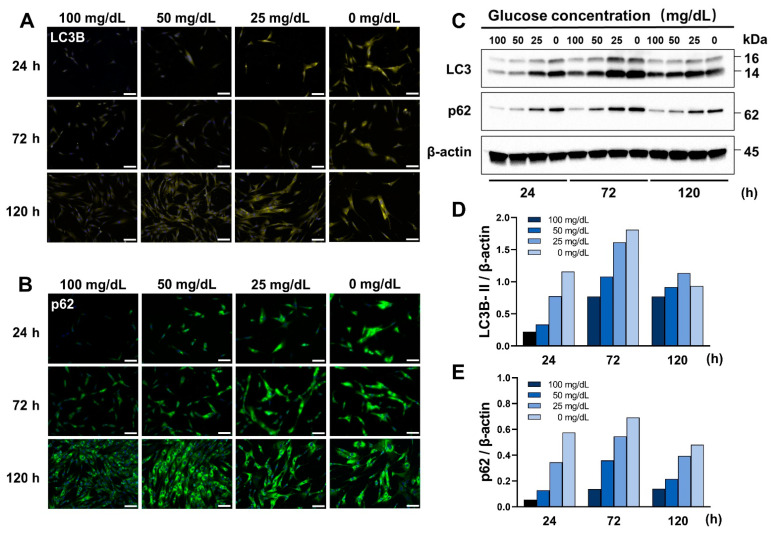
Low glucose conditions induce autophagy in HGnFs. (**A**,**B**) Synthesis of autophagy-related proteins (Total-LC3B and p62) by HGnFs under low glucose conditions, after 24, 72, and 120 h of incubation. The HGnFs were photographed after incubation with fluorescently labeled secondary antibodies, and nuclei were stained with DAPI. (**C**–**E**) The expression of LC3B and p62 was examined using western blot analysis; the data were obtained using protein extracts of these cells with antibodies against the indicated proteins with β-actin as a loading control. The samples derived from the same experiment and their corresponding gels/blots were processed in parallel. (Scale bars: 100 μm).

To investigate the effect of low glucose on the autophagy of HGnFs, Western blot and immunofluorescence were employed. As shown in Figure 3, after treatment with four different glucose concentrations (100, 50, 25, and 0 mg/dL) for 24, 72, and 120 h, the expression of LC3B and p62 as autophagy-related proteins was significantly increased in the treatment group compared to that in the control group.

### 3.4. NAC Administration Inhibited ROS Formation

**Figure 4:**
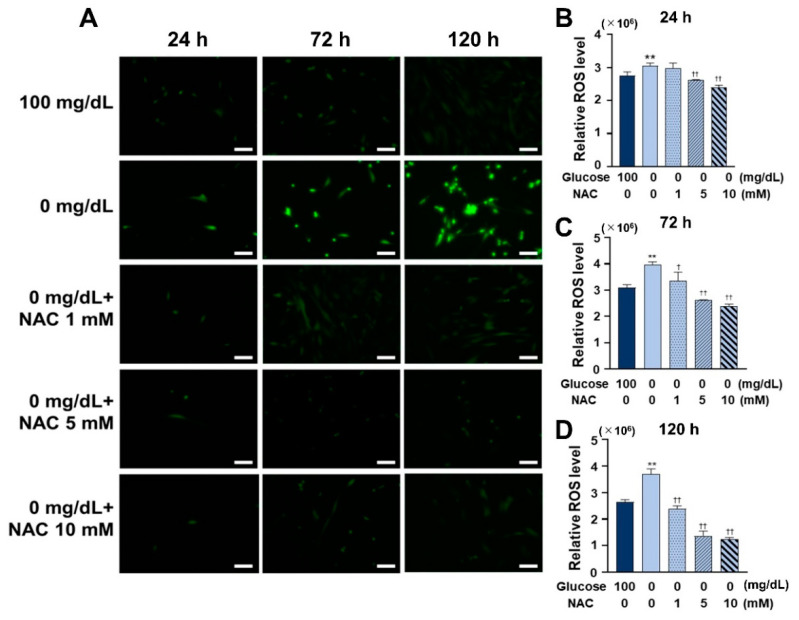
Effect of NAC on ROS formation and anti-ROS ability under low glucose conditions. (**A**) The total ROS of the HGnFs was stained and photographed under a fluorescence microscope after adding 1, 5, and 10 mM NAC and incubating for 24, 72, and 120 h. (**B**–**D**) Total ROS levels after 24, 72, and 120 h of incubation with 1, 5, and 10 mM of NAC were measured using a fluorescent microplate reader, and the results were modified relative to 100 mg/dL (control group). (Scale bars: 100 μm; a significant increase compared with the control was described as ** *p* < 0.01; a significant decrease compared with the 0 mg/dL was described as † *p* < 0.05, †† *p* < 0.01).

The effects of NAC on glucose starvation and ROS formation were measured after culturing the cells for 24, 72, and 120 h. As shown in Figure 4, ROS formation was inhibited significantly in all NAC-treated groups (1, 5, and 10 mM), with the greatest inhibition observed at a NAC concentration of 10 mM.

### 3.5. Administration of NAC Rescued Cell Viability and Inhibited the Expression of Inflammatory Cytokines under Glucose Starvation Conditions

**Figure 5:**
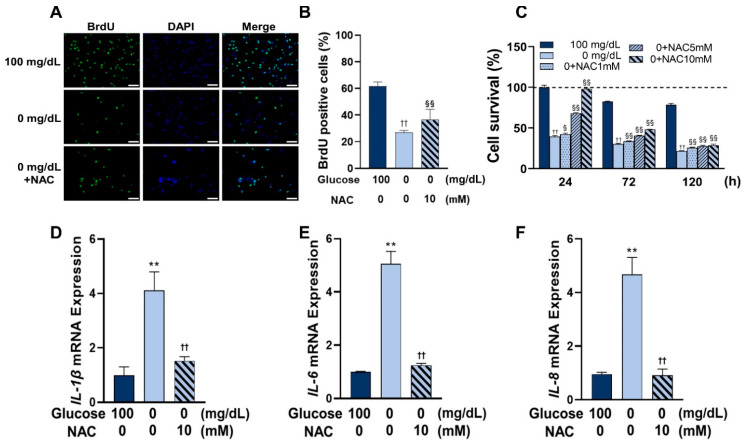
NAC rescued the cellular viability and reduced inflammation in HGnFs induced by low glucose conditions. (**A**,**B**) Representative immunofluorescence images of BrdU were incorporated into DNA after the addition of 10 mM NAC, and the nuclei were stained by DAPI. The quantitative analysis of BrdU-positive cells was presented as mean ± SD of three independent experiments. (**C**) Cell survival after the addition of 10 mM NAC was measured using an MTT assay. The average of the absorbance of each group at 0 h was used as 100% to calculate the relative results. (**D**–**F**) The mRNA expression of inflammatory cytokines, such as IL-1β, IL-6, and IL-8, was determined using real-time PCR in stimulated HGnFs incubated for 72 h. (Scale bars: 100 μm; a significant increase compared with the control was described as ** *p* < 0.01; a significant increase compared with the 0 mg/dL group was described as § *p* < 0.05, §§ *p* < 0.01; a significant decrease compared with the group of 0 mg/dL was described as †† *p* < 0.01.).

As shown in Figure 5, cell proliferation and survival were also measured under glucose starvation conditions, with a significant rescue of cell proliferation and survival by NAC and the best rescue occurring at a NAC concentration of 10 mM. Moreover, the administration of NAC significantly inhibited the gene expression of IL-1β, IL-6, and IL-8 as inflammatory cytokines, under glucose starvation conditions after 72 h of incubation.

### 3.6. NAC Treatment Suppressed the Process of Autophagy and the ROS-Akt/ERK Signaling Pathway under Glucose Starvation Conditions

**Figure 6:**
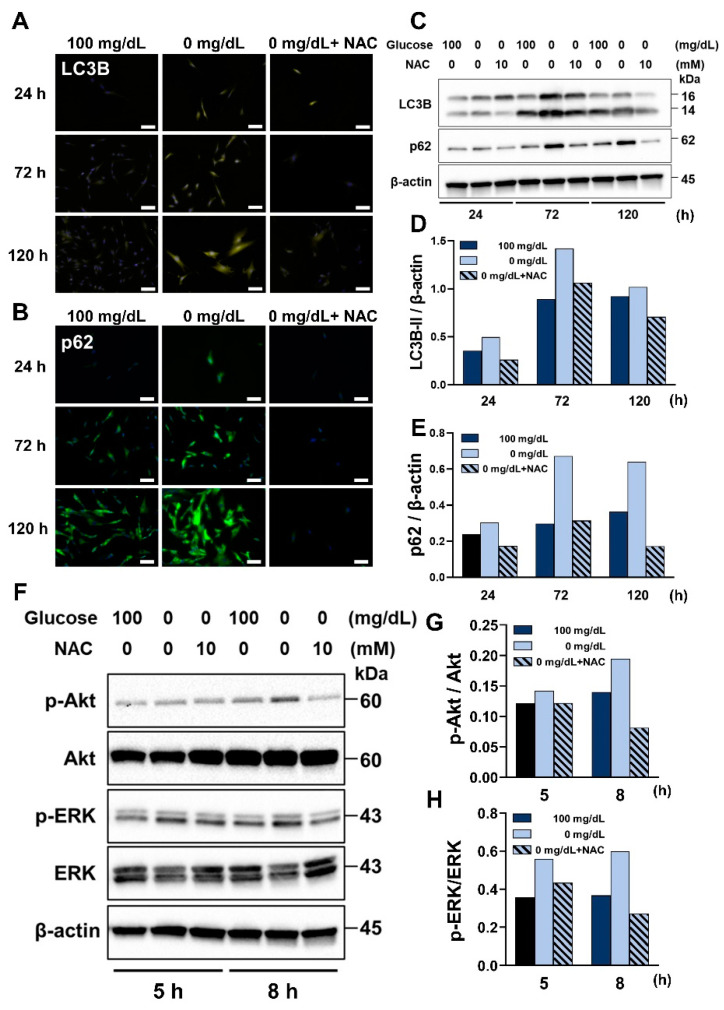
NAC prevented the induction of autophagy in the HGnFs under low glucose conditions and inhibited the phosphorylation of the ROS-Akt/ERK signaling pathway. (**A**,**B**) Synthesis of autophagy-related proteins (Total-LC3B and p62) in the HGnFs after 24, 72, and 120 h of incubation under low glucose conditions following the addition of NAC. The nuclei were stained with DAPI. (**C**–**E**) LC3B and p62 expression were analyzed using western blot, performed on protein extracts from these cells with antibodies against the described proteins and β-actin serving as a loading control. (**F**–**H**) The protein expression of the ROS-Akt/ERK signaling pathway was evaluated using western blotting analysis and quantified using ImageJ. Western blot analysis was performed on protein extracts of these cells with antibodies against the indicated proteins and β-actin as a loading control. The samples derived from the same experiment and the gels/blots were processed in parallel. The expression levels of Akt, p-Akt, ERK, and p-ERK were measured by densitometric analysis using ImageJ. (Scale bar: 100 μm.).

As shown in Figure 6A–E, the effects of NAC treatment under glucose starvation on autophagy-related protein expression in HGnFs were analyzed using Western blot and immunofluorescence staining. LC3B and p62 expression significantly was reduced in the NAC-treated groups at 24, 72, and 120 h compared to that in the control group. Additionally, as shown in Figure 6F,G, after 5 and 8 h of stimulation, glucose starvation conditions significantly increased the phosphating levels of ERK and Akt, and this increase was significantly reduced by NAC administration.

## 4. Discussion

In this study, the mechanisms of cellular damage under low glucose conditions were investigated. The results showed that low glucose conditions induced ROS formation, which enhanced inflammation and autophagy in HGnFs. After blocking ROS formation, both inflammation and autophagy were controlled, and cellular activity was recovered.

Although many reports suggest that high glucose conditions associated with diabetes induce ROS formation [16], we demonstrated that low glucose conditions also induce ROS formation. HO-1 and SOD are intracellular anti-ROS enzymes that act as cytoprotective agents by acting as a neutralizer to ROS [17]. High glucose conditions-induced ROS promote the expression of HO-1 and SOD to counteract the damaging effects of ROS on cells. In this study, we observed a similar phenomenon, where elevated ROS expression was accompanied by enhanced HO-1 and SOD expression. At high glucose concentrations, mitochondria are the main source of ROS because high glucose concentrations increase the metabolic input to the mitochondria, overwhelming the ETC and leading to mitochondrial hyperpolarization and excessive ROS production [18]. Low glucose environments may also have a similar mechanism for inducing ROS. ROS can impair normal cellular function. Our previous study showed that low glucose conditions also impair cellular functions, such as proliferation and migration, in HGnFs [9]. In this study, we used different assays to detect ROS production, successfully confirming previous findings while investigating the expression of anti-ROS enzymes HO-1 and SOD.

In this study, a significantly reduced proportion of BrdU bound to DNA was observed in the groups with glucose concentrations of 25 and 0 mg/dL, indicating a reduced proliferation capacity at low glucose concentrations. In addition, a progressive decrease in cell viability at lower glucose concentrations was found. These results suggest that low concentrations of glucose can induce ROS by weakening the ability to resist ROS and impairing cell viability.

ROS play an important role in the progress of inflammation [8]. Enhanced ROS formation at the site of inflammation leads to tissue dysfunction and damage [19]. Studies have shown that inflammation is deeply involved in the gingival wound healing process, and excessive inflammation delays this process [20,21]. Some studies have shown that high glucose conditions induce high expression of inflammatory cytokines and impair normal cell function, as in the case of differentiation of PDLSCs [22]. In this study, low glucose conditions also induced inflammation by the overexpression of inflammatory cytokines. These results may provide new insights into the mechanisms of inflammation that occur in wound tissue.

Autophagy is a process by which cells actively degrade their own cellular contents, and this process is important for balancing energy sources in response to nutrient deficiency stress [11]. It plays an important role in the removal of damaged organelles, such as mitochondria, the elimination of intracellular pathogens, and the removal of misfolded proteins [23]. Recent studies have suggested that autophagy induces inflammatory cytokines and ROS formation [24]. There is a close relationship between ROS, inflammation, and autophagy. ROS can directly trigger autophagy, and autophagy can induce the production of inflammatory cytokines [25]. In this study, we found that low glucose conditions induce autophagy in HGnFs. Interestingly, even in the 100 mg/dL glucose concentration group with sufficient nutritional conditions, autophagy could be observed at 72 and 120 h, which suggests that HGnFs have more active autophagic activity than other cells.

To further explore the role of ROS in low glucose conditions, we used NAC, a widely used antioxidant that can act directly as a scavenger of free radicals, especially oxygen radicals [26]. NAC is a powerful antioxidant, and it has been recommended as a potential treatment option for different disorders resulting from the generation of free oxygen radicals [27]. NAC was used to inhibit ROS formation in low glucose conditions and observe the progress of inflammation and autophagy induced under low glucose conditions. The effective concentration of NAC varies depending on the cell type. Therefore, first, the antioxidant effect of NAC was tested using three concentrations (1, 5, and 10 mM) to determine an ideal concentration, and the results demonstrated that all three concentrations were effective in reducing ROS formation. Then, the most effective concentration, 10 mM, was chosen for the subsequent experiments. It was then observed that cell survival and the amount of BrdU-bonded DNA also increased on NAC treatment, suggesting that the reduced cellular activity at low glucose concentrations was caused by ROS, and this ROS-induced oxidative damage can be rescued by NAC.

Next, the expression of inflammatory factors following NAC treatment was investigated. A reduction in the mRNA expression of IL-1β, IL-6, and IL-8 was identified. Additionally, the expression of autophagy-related proteins, LC3 and p62, was reduced after NAC treatment. Study has shown that NAC inhibited the JNK signaling pathway, which suppressed inflammation and autophagy in ischemia-reperfusion injured mouse cells [28]. This is similar to our results, in that, NAC inhibited low glucose condition-induced inflammation and autophagy. Studies have shown that the phagocytosis of cytoplasmic components, including cytosolic proteins and organelles, is induced by autophagosomes during autophagy. At the same time, a cytosolic form of LC3-I was conjugated to phosphatidylethanolamine to form LC3-II, which was recruited to autophagosomal membranes. Autophagosomes fused with lysosomes to form autolysosomes, and intra-autophagosomal components were degraded by autolysosomal hydrolases [29]. Thus, the expression of LC3-I can be considered to represent the pre-process of autophagy, whereas the expression of LC3-II represents the overall process of autophagic activity and autophagy. In this study, an increase in LC3-I and a decrease in LC3-II expression were observed, which suggests that NAC inhibits the conversion of the lipid modification process from LC3-I to LC3-II. In other words, ROS may be important for the pre-process of autophagy in gingival tissue.

An increasing number of studies have provided information that Akt and ERK signaling pathways can collaborate to maintain cellular function in a cell type-dependent manner [10]. These two signaling pathways are cross-linked at many sites, and either signaling pathway can enhance or inhibit signaling in the other. Both pathways can be activated by ROS and perform many physiological functions, such as autophagy. Phosphorylation of ERK is necessary for the autophagic process [30], and ERK pathway inhibitors can be used to inhibit autophagy [31]. In this study, NAC inhibited the phosphorylation of ERK pathways activated by ROS, which may suggest that ROS is required for ERK phosphorylation for the induction of autophagy in low glucose conditions. Akt activation upregulates intracellular ROS levels by inhibiting ROS clearance [32]. Akt also inhibits cell proliferation by promoting mitochondrial biogenesis to upregulate ROS and by activating the DNA damage response pathway; this inhibitory mechanism can be rescued by NAC [33]. Additionally, the phosphorylation of Akt could induce autophagy, thus promoting gingival wound healing [34]. We recognized that NAC inhibited the phosphorylation of Akt, which may be related to the inhibition of the process of autophagy, restoration of cell viability and reduced levels of inflammation observed in this study.

Clinically, it is considered that after trauma or periodontal surgery, the blood supply to periodontal tissue is lower than that of healthy tissue due to damaged blood vessels, resulting in the tissue experiencing a state of glucose starvation. For example, when free gingival autografting (FGG) is performed, control of the blood supply and inflammation of the free tissue is considered critical to the success of the free soft tissue graft [35]. When the blood supply is not restored, cells from the free soft tissue (mainly gingival fibroblasts) experience a stressful microenvironment characterized by low glucose concentrations. In this study, we demonstrated that low glucose concentrations lead to the accumulation of ROS in HGnFs and result in decreased cell viability and inflammation. The findings of this study provide a new insight of the importance of soft tissue management in periodontal surgical procedures.

In this study, the low nutrient microenvironment in the periodontal-wound tissue was simulated by using a low glucose environment, as glucose is one of the most important components of all nutrients. However, the limitation of this study is that several other factors in the periodontal-wound microenvironment, such as bacterial infection and serum or blood oxygen levels, were not simulated due to its in vitro design. In addition, we opted for an in vitro design because an in vivo design would mean that the animals themselves may die of hypoglycemia and the experimental process may be discontinued if blood glucose levels are artificially lowered in animal experiments; therefore, we employed culture experiments. Furthermore, other antioxidants such as Resveratrol and Quercetin have different mechanisms for blocking ROS compared to NAC. Resveratrol and Quercetin induce an increase in mitochondrial respiration, while NAC enhances more non-mitochondrial respiration in circumstances of consumption of less oxygen [36]. Therefore, further studies are needed to fully elucidate these mechanisms while considering these variables.

## 5. Conclusions

**Figure 7:**
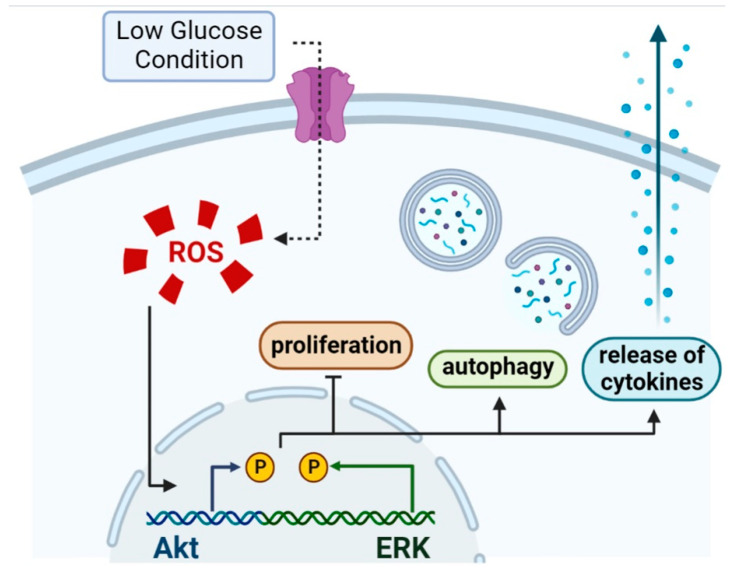
The low glucose environment could increase ROS accumulation. Increased ROS allows for phosphorylation of the Akt signal pathway and ERK pathway, which inhibits cell proliferation, induces cellular autophagy, and increases the expression of inflammatory cytokine.

In conclusion, it was found, for the first time, that a low glucose environment could induce ROS formation and thus activates the Akt and ERK signaling pathways, which lead to impaired cellular viability and the induction of inflammation and autophagy is shown in Figure 7. This process could be rescued by NAC. This finding may provide new insights into the mechanisms of wound healing after periodontal surgery under glucose starvation environment.

## Data Availability

The datasets generated during and/or analyzed during the current study are available from the corresponding author on reasonable request.

## References

[1] Galant A.L., Kaufman R.C., Wilson J.D. (2015). Glucose: Detection and Analysis. Food Chem..

[2] Verdonk C.A., Rizza R.A., Gerich J.E. (1981). Effects of Plasma Glucose Concentration on Glucose Utilization and Glucose Clearance in Normal Man. Diabetes.

[3] Rossetti L., Giaccari A., DeFronzo R.A. (1990). Glucose Toxicity. Diabetes Care.

[4] Wolff S.P., Dean R.T. (1987). Glucose Autoxidation and Protein Modification. The Potential Role of “autoxidative Glycosylation” in Diabetes. Biochem. J..

[5] Lee T.S., Saltsman K.A., Ohashi H., King G.L. (1989). Activation of Protein Kinase C by Elevation of Glucose Concentration: Proposal for a Mechanism in the Development of Diabetic Vascular Complications. Proc. Natl. Acad. Sci. USA.

[6] Rosca M.G., Mustata T.G., Kinter M.T., Ozdemir A.M., Kern T.S., Szweda L.I., Brownlee M., Monnier V.M., Weiss M.F. (2005). Glycation of Mitochondrial Proteins from Diabetic Rat Kidney Is Associated with Excess Superoxide Formation. Am. J. Physiol.-Ren. Physiol..

[7] Yang H., Jin X., Lam C.W.K., Yan S.K. (2011). Oxidative Stress and Diabetes Mellitus. Clin. Chem. Lab. Med..

[8] Zhang J., Wang X., Vikash V., Ye Q., Wu D., Liu Y., Dong W. (2016). ROS and ROS-Mediated Cellular Signaling. Oxid. Med. Cell. Longev..

[9] Li R., Kato H., Taguchi Y., Umeda M. (2022). Intracellular Glucose Starvation Affects Gingival Homeostasis and Autophagy. Sci. Rep..

[10] Dent P. (2014). Crosstalk between ERK, AKT, and Cell Survival. Cancer Biol. Ther..

[11] Rabinowitz J.D., White E. (2010). Autophagy and Metabolism. Science.

[12] Bullon P., Cordero M.D., Quiles J.L., Ramirez-Tortosa M.D.C., Gonzalez-Alonso A., Alfonsi S., García-Marín R., de Miguel M., Battino M. (2012). Autophagy in Periodontitis Patients and Gingival Fibroblasts: Unraveling the Link between Chronic Diseases and Inflammation. BMC Med..

[13] Greabu M., Giampieri F., Melescanu Imre M., Mohora M., Totan A., Pituru S.M., Ionescu E. (2020). Autophagy, One of the Main Steps in Periodontitis Pathogenesis and Evolution. Molecules.

[14] Kim W.J., Park S.Y., Kim O.S., Park H.S., Jung J.Y. (2022). Autophagy Upregulates Inflammatory Cytokines in Gingival Tissue of Patients with Periodontitis and Lipopolysaccharide-Stimulated Human Gingival Fibroblasts. J. Periodontol..

[15] Matsuzawa-Ishimoto Y., Hwang S., Cadwell K. (2018). Autophagy and Inflammation. Annu. Rev. Immunol..

[16] Buranasin P., Mizutani K., Iwasaki K., Mahasarakham C.P.N., Kido D., Takeda K., Izumi Y. (2018). High Glucose-Induced Oxidative Stress Impairs Proliferation and Migration of Human Gingival Fibroblasts. PLoS ONE.

[17] Zheng Y., Liu Y., Ge J., Wang X., Liu L., Bu Z., Liu P. (2010). Resveratrol Protects Human Lens Epithelial Cells against H2O2- Induced Oxidative Stress by Increasing Catalase, SOD-1, and HO-1 Expression. Mol. Vis..

[18] Yu T., Jhun B.S., Yoon Y. (2011). High-Glucose Stimulation Increases Reactive Oxygen Species Production through the Calcium and Mitogen-Activated Protein Kinase-Mediated Activation of Mitochondrial Fission. Antioxid. Redox Signal..

[19] Kashiwagi Y., Takedachi M., Mori K., Kubota M., Yamada S., Kitamura M., Murakami S. (2016). High Glucose-Induced Oxidative Stress Increases IL-8 Production in Human Gingival Epithelial Cells. Oral Dis..

[20] Haekkinen L.A.R.I., Uitto V.J., Larjava H. (2000). Cell Biology of Gingival Wound Healing. Periodontol.

[21] Young A., McNaught C.E. (2011). The Physiology of Wound Healing. Surgery.

[22] Kato H., Taguchi Y., Tominaga K., Kimura D., Yamawaki I., Noguchi M., Yamauchi N., Tamura I., Tanaka A., Umeda M. (2016). High Glucose Concentrations Suppress the Proliferation of Human Periodontal Ligament Stem Cells and Their Differentiation Into Osteoblasts. J. Periodontol..

[23] Mizushima N. (2007). Autophagy: Process and Function. Genes Dev..

[24] Herzig S., Shaw R.J. (2018). AMPK: Guardian of Metabolism and Mitochondrial Homeostasis. Nat. Rev. Mol. Cell Biol..

[25] Scherz-Shouval R., Elazar Z. (2007). ROS, Mitochondria and the Regulation of Autophagy. Trends Cell Biol..

[26] Aldini G., Altomare A., Baron G., Vistoli G., Carini M., Borsani L., Sergio F. (2018). N-Acetylcysteine as an Antioxidant and Disulphide Breaking Agent: The Reasons Why. Free Radic. Res..

[27] Kelly G.S. (1998). Clinical Applications of N-Acetylcysteine. Altern. Med. Rev..

[28] Wang C., Chen K., Xia Y., Dai W., Wang F., Shen M., Cheng P., Wang J., Lu J., Zhang Y. (2014). N-Acetylcysteine Attenuates Ischemia-Reperfusion-Induced Apoptosis and Autophagy in Mouse Liver via Regulation of the ROS/JNK/Bcl-2 Pathway. PLoS ONE.

[29] Tanida I. (2011). Autophagy Basics. Microbiol. Immunol..

[30] Martinez-Lopez N., Athonvarangkul D., Mishall P., Sahu S., Singh R. (2013). Autophagy Proteins Regulate ERK Phosphorylation. Nat. Commun..

[31] Corcelle E., Djerbi N., Mari M., Nebout M., Fiorini C., Fénichel P., Hofman P., Poujeol P., Mograbi B. (2007). Control of the Autophagy Maturation Step by the MAPK ERK and P38: Lessons from Environmental Carcinogens. Autophagy.

[32] Zhao Y., Hu X., Liu Y., Dong S., Wen Z., He W., Zhang S., Huang Q., Shi M. (2017). ROS Signaling under Metabolic Stress: Cross-Talk between AMPK and AKT Pathway. Mol. Cancer.

[33] Polytarchou C., Hatziapostolou M., Yau T.O., Christodoulou N., Hinds P.W., Kottakis F., Sanidas I., Tsichlis P.N. (2020). Akt3 Induces Oxidative Stress and DNA Damage by Activating the NADPH Oxidase via Phosphorylation of P47phox. Proc. Natl. Acad. Sci. USA.

[34] Kominato H., Takeda K., Mizutani K., Mikami R., Kido D., Buranasin P., Saito N., Takemura S., Nakagawa K., Nagasawa T. (2022). Metformin Accelerates Wound Healing by Akt Phosphorylation of Gingival Fibroblasts in Insulin-Resistant Prediabetes Mice. J. Periodontol..

[35] Dibart S. (2017). Free Gingival Autograft. Pract. Periodontal Plast. Surg..

[36] Orihuela-Campos R.C., Tamaki N., Mukai R., Fukui M., Miki K., Terao J., Ito H.-O. (2015). Biological Impacts of Resveratrol, Quercetin, and N-Acetylcysteine on Oxidative Stress in Human Gingival Fibroblasts. J. Clin. Biochem. Nutr..

